# Threshold Responses to Soil Moisture Deficit by Trees and Soil in Tropical Rain Forests: Insights from Field Experiments

**DOI:** 10.1093/biosci/biv107

**Published:** 2015-08-31

**Authors:** Patrick Meir, Tana E. Wood, David R. Galbraith, Paulo M. Brando, Antonio C. L. Da Costa, Lucy Rowland, Leandro V. Ferreira

**Affiliations:** Patrick Meir is affiliated with the Research School of Biology at Australian National University, in Canberra, and with the School of Geosciences at the University of Edinburgh, in the United Kingdom. Tana E. Wood is affiliated with the US Department of Agriculture Forest Service's International Institute of Tropical Forestry, in Rio Piedras, Puerto Rico, and with the Fundación Puertorriqueña de Conservación, in San Juan, Puerto Rico. David R. Galbraith is affiliated with the School of Geography at the University of Leeds, in the United Kingdom. Paulo M. Brando is with the Instituto Pesquisa Ambiental Amazonia, in Belém, Brazil. Antonio C. L. da Costa is affiliated with the Universidade Federal de Para, in Belém, Brazil. Lucy Rowland is with the Research School of Biology at Australian National University, in Canberra. Leandro V. Ferreira is affiliated with the Museu Paraense Emílio Goeldi, in Belém, Brazil.

**Keywords:** drought, tropical rain forest, tree mortality, physiology, soil respiration

## Abstract

Many tropical rain forest regions are at risk of increased future drought. The net effects of drought on forest ecosystem functioning will be substantial if important ecological thresholds are passed. However, understanding and predicting these effects is challenging using observational studies alone. Field-based rainfall exclusion (canopy throughfall exclusion; TFE) experiments can offer mechanistic insight into the response to extended or severe drought and can be used to help improve model-based simulations, which are currently inadequate. Only eight TFE experiments have been reported for tropical rain forests. We examine them, synthesizing key results and focusing on two processes that have shown threshold behavior in response to drought: (1) tree mortality and (2) the efflux of carbon dioxdie from soil, soil respiration. We show that: (a) where tested using large-scale field experiments, tropical rain forest tree mortality is resistant to long-term soil moisture deficit up to a threshold of 50% of the water that is extractable by vegetation from the soil, but high mortality occurs beyond this value, with evidence from one site of increased autotrophic respiration, and (b) soil respiration reaches its peak value in response to soil moisture at significantly higher soil moisture content for clay-rich soils than for clay-poor soils. This first synthesis of tropical TFE experiments offers the hypothesis that low soil moisture–related thresholds for key stress responses in soil and vegetation may prove to be widely applicable across tropical rain forests despite the diversity of these forests.

**The gross exchanges of mass and energy in** tropical forests are larger than for any other terrestrial ecosystem (Bonan 2008). Like all biophysical systems they are sensitive to climate, and because of their large extent, small fractional changes in these gross fluxes may be globally significant for climate. Given the substantial use of tropical forests by and to humanity, their large geographical extent, species diversity, biomass density, and their high rates of metabolism, improved understanding of their responses to climate change is needed. These responses may be large and rapid if ecological thresholds are passed (Reichstein et al. 2013), and the risk during this century may be particularly high for tropical ecosystems (Mora et al. 2013).

Although spatially variable, climate-change scenarios for tropical forest regions, including tropical rain forests, emphasize the likelihood of future warming and an increased frequency of extremes of temperature and rainfall, especially in relation to increased dry-season length (Fu et al. 2013). In the last two decades, examples of extremes in rainfall have been observed pantropically, including exceptional El Niño Southern Oscillation events, two once-in-a-century drought events across Amazonia in 2005 and 2010, and recent extended declines in dry season rainfall (Marengo et al. 2011, Fu et al. 2013). Two important processes in forested ecosystems that may change substantially in response to altered moisture conditions, even in the absence of fire, are tree mortality and the emission of carbon dioxide (CO_2_) from soil, usually referred to as soil respiration (Davidson et al. 2006, Allen et al. 2010). Changes in both have the capacity to cause globally significant alterations to ecosystem functioning and atmospheric CO_2_ concentration this century. However, the understanding needed to constrain tropical forest responses to projected changes in rainfall remains limited (Meir and Woodward 2010).

Observational studies have provided insights into the response by tropical rain forests to reductions in soil moisture availability. Early flux measurements demonstrated varying degrees of daily and seasonal sensitivity to soil moisture in the carbon cycle of tropical rain forest (Grace et al. 1995, Malhi et al. 1998, Araujo et al. 2002, Saleska et al. 2003, Kumagai and Kume 2012). Plot-based observations of tree growth, recruitment and mortality have also identified high sensitivity to short-term severe drought in all major tropical regions (Meir and Grace 2005, Phillips et al. 2010). However, a lack of detailed component-scale flux data has limited process-level interpretation, especially below ground (Meir et al. 2008). Forward modeling of vegetation and soil processes for tropical forests against future climate scenarios remains in its infancy, and recent analyses using dynamic global vegetation models (used, e.g., in Earth system models) have highlighted both model insensitivity to moisture deficit in some processes such as tree mortality, and model oversensitivity to temperature in others such as respiration (Galbraith et al. 2010, Powell et al. 2013). There also remain gaps in understanding of plant physiological responses to drought, associated processes such as insect or pathogen attack, and the differing effects of the extremity and the duration of the climatic drivers (Anderegg et al. 2013, McDowell et al. 2013). Globally, there is a paucity of relevant data to inform vegetation models on these questions, and although the effects of forest loss from drought could be large (Reichstein et al. 2013), nowhere is this data gap more obvious than in the tropics, especially with respect to the few rainfall manipulation experiments that have been implemented to date in tropical rain forests. We examine those experiments here.

Experimental reduction of soil moisture is achieved in forests by deflecting the rainfall that penetrates a canopy (*throughfall*) away from treatment plots using a drainage structure installed 1–2 meters (m) above the forest floor ­(figure 1). This *throughfall exclusion* (TFE) approach complements studies of natural drought because soil moisture can be altered beyond normal ranges, independently of other climate drivers, and at large or small scales. This is valuable in circumstances in which ecological treatment responses may become nonlinear or alter qualitatively over different time scales (Leuzinger et al. 2011, Wu et al. 2011). Eight TFE studies have been implemented in tropical rain forests in recent years (table 1). They vary in plot size, treatment duration and measurements, and this limits comprehensive detailed comparison, so here we consider the responses of two widely measured processes that have shown threshold behavior in response to low soil moisture availability and have the potential to significantly alter ecosystem functioning: tree mortality and soil respiration. We combine data sets from different studies (box 1) to test the broad hypotheses that: (a) the increase in tropical rain forest tree mortality during extended soil moisture deficit is nonlinear with respect to soil moisture deficit, and this response function is similar across sites, and (b) soil texture explains the observed differences in the responses by soil respiration to reduced soil moisture. We also consider how the TFE approach is useful for developing ecological insight into ecosystem–environment responses over multiple timescales.

**Figure 1.**
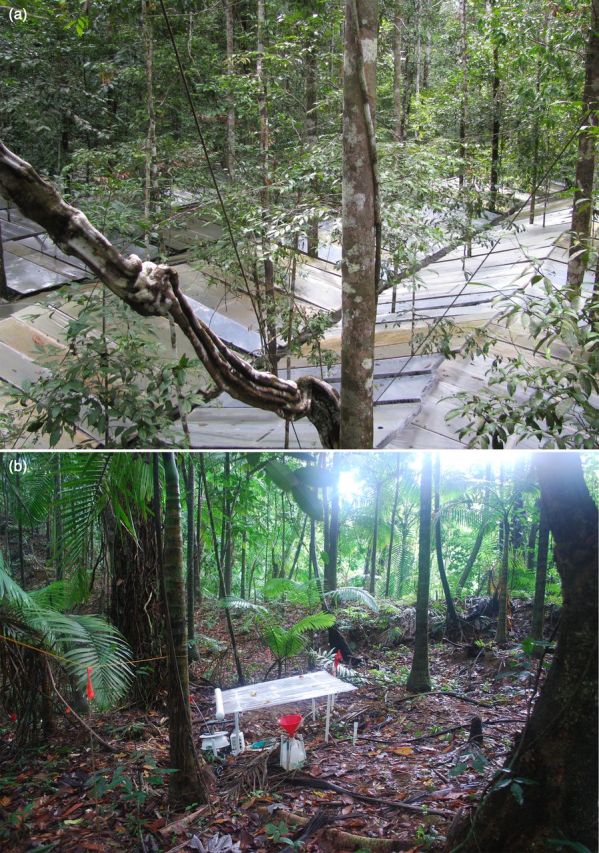
Large- and small-scale throughfall exclusion (TFE) structures in tropical rain forest, placed 0.5–2 meters aboveground level. (a) Caxiuanã National Forest, Pará, Brazil (treatment size: 1 hectare). (b) Luquillo Experimental Forest, Puerto Rico (treatment size: 1.5 square meters).

**Table 1.** Site details for the three ecosystem-scale throughfall exclusion (TFE) experiments in tropical rain forest (CAX, TAP, SUL) and five smaller-scale tropical rain forest TFE experiments (FAZ, OSA, LEF_ridge_, LEF_slope_, LEF_valley_).

Site ID	Elevation (in meters)	MAP	MAT	Soil type	Soil depth (in meters)	Aboveground biomass (in megagrams of carbon per hectare)	Percentage rain excluded	TFE length (in months)	Plot size (in square meters)	*R*_s_
CAX^a^	15	2300	26	Oxisol	10–15	215	50	120	10 000	4.3
TAP^b^	150	2000	27	Oxisol	>80	270	50	72	10 000	2.6
SUL^c^	1050	2900	21	Nitisol	>4	300	50–80	24	1600	3.2
FAZ^d^	100^g^	1800	26	Oxisol	>18	260	50	12	100	2.7
OSA^e^	50	5000	27	Ultisol	n/a	180	50	12	5.76	3.0
LEF_ridge_^f^	350	3500	23	Ultisol	33 m	312	100	3	1.54	3.6
LEF_slope_^f^	350	3500	23	Ultisol	20 m	186	100	3	1.54	3.4
LEF_valley_^f^	350	3500	23	Ultisol	20 m	121	100	3	1.54	4.1

*Note:* Detailed locations are in the reference citations. Elevation is in meters (m) above sea level. Abbreviations: CAX, Caxiuanã National Forest Reserve, Pará, Brazil; FAZ, Fazenda Vitoria, Pará, Brazil; LEF, Luquillo Experimental Forest, Puerto Rico; MAP, mean annual precipitation (in millimeters); MAT, the mean annual temperature (degrees Celsius); OSA, Osa Peninsula, Costa Rica; *R*_s_, mean annual soil carbon dioxide efflux from undisturbed forest (in micromols CO_2_ per square meter per second); SUL, Pono Valley, Lore Lindu National Park, Central Sulawesi, Indonesia; TAP, Tapajós National Forest Reserve, Para Brazil. ^a^Meir et al. 2009. ^b^Brando et al. 2008. ^c^Schuldt et al. 2011, Moser et al. 2014. ^d^Cattanio et al. 2002. ^e^Cleveland et al. 2010. ^f^Wood and Silver 2012, Scatena and Lugo 1995. ^g^Estimated.

**Box 1. Analysis of combined data sets from multiple studies.**

Large-scale TFE is required to examine experimentally how soil moisture deficit affects ecosystem-level metrics of ecosystem function such as tree mortality. At all three such tropical rain forest experiments, CAX, TAP, and SUL, there was no significant change in tree mortality for the first 2 years of TFE, but whilst the experiment at SUL was terminated after 2 years, mortality increased substantially at CAX and TAP from year 3 onward (figure 2a; da Costa et al. 2010, Moser et al. 2014). Tree mortality and soil moisture data at CAX and TAP were combined and analysed to examine hypothesis (a). Soil moisture availability was expressed as relative extractable water (REW) over the top 3 m of soil. REW is defined as the fraction of the maximum extractable water content (plant available water, *PAW*_max_) for each soil layer. *PAW*_max_ refers to the soil moisture available between field capacity (*θ*_fc_), typically taken to be –33 kilopascals (kPa) and wilting point (*θ*_w_), typically taken to be –1500 kPa. However, it is very difficult to obtain accurate values of *θ*_w_ and *θ*_fc._ These can be derived from soil water characteristic curves where available, or from the use of pedotransfer functions, but such functions are highly uncertain and the actual wilting point (soil water potential at which water uptake ceases) is rarely known. Here, we use a very simple metric to calculate REW, in which we set *θ*_w_ to be equal to the minimum volumetric water content (VWC) value observed (*θ*_min_) and *θ*_fc_ to be equal to the observed maximum monthly mean VWC. Such a simple approach is consistent with previous studies (Granier et al. 2000, Nepstad et al. 2007) and provides us with a comparable metric with which to estimate plant water availability across plots. Therefore, for each soil layer *i*, REW is estimated as

$$\text{REW}=\frac{\theta -\theta_{\min}}{\theta_{\max}-\theta_{\min}}$$

in which *θ* is the actual volumetric water content, *θ*_min_ is the minimum volumetric water content and *θ*_max_ is the maximum monthly mean VWC. We use detailed soil profile VWC measurements and validated model results (Fisher et al. 2006, 2007, Nepstad et al. 2007), weighting layer-specific REW values by soil layer depths to calculate REW for the top 3 m. We computed annual values of REW for each year in the control and treatment plots and related these to annual values of stem and biomass mortality. The relationship between REW and mortality was examined using linear and nonlinear fits, including piecewise regression, using the statistical package R (R 2.14.2, R-project software, *www.r-project.org*, R 2.14.2). The vulnerability to mortality of the most well-represented taxonomic groups at each site was also examined by comparing mortality between treatment and control plots (da Costa et al. 2010).

Soil processes in tropical forests have been studied using TFE treatments varying in size from 1.5 m^2^ to 1 ha, and in duration from seasonal TFE to a decade-long experiment (table 1). For the soil respiration (*R*_s_) analysis (hypothesis (b)), site level data were collated from each: mean annual precipitation (MAP), mean annual temperature (MAT), volumetric soil moisture content (percent), soil texture (percent clay, silt, and sand), available nutrient pools (total phosphorus, total nitrogen, available phosphorous, nitrate and ammonium) together with mean surface emissions of CO_2_ (*R*_s_). As most TFE treatments were short in duration (3 months–2 years), analysis of combined data sets was focused on the short-term phase of soil moisture reduction. General linear regression was used to examine single and multivariate relationships among soil moisture content, soil texture and soil gas emission metrics during soil moisture deficit. The threshold soil moisture content at which maximum *R*_s_ was observed was analysed using a dataset expanded to incorporate a wider range of soil texture values by also including observational studies from tropical rainforest (listed in appendix S1).

## Vegetation responses to ecosystem-scale rainfall exclusion

The sudden reduction in live biomass resulting from widespread drought-induced tree mortality has received much attention in the last 5–10 years (Breshears et al. 2005, Allen et al. 2010, McDowell et al. 2011). There is evidence that drought and warming events have already led to notable forest dieback events globally, with the majority of reports from northern hemisphere temperate zones (Allen et al. 2010). Even in the absence of fire, severe droughts have also been shown to cause very large increases in tree mortality across the tropics (Meir and Grace 2005) with sufficient data in one instance to indicate a switch in the sign of estimated aboveground carbon gain to carbon emission (Phillips et al. 2009). However, the causes of drought-related tree mortality remain unclear. It has been argued that failure in the transport of water to the canopy, or failure in the supply of sufficient carbohydrate to metabolising tissues, may lead to death via desiccation, carbon ‘starvation’ of metabolism (including osmoregulatory processes), or alternatively through weakened defences to pest and pathogen attack (e.g., McDowell et al. 2011, McDowell et al. 2013, O'Brian et al. 2014). Current analysis, mainly from temperate-zone forests, supports all three explanations to varying degrees and this lack of clarity hampers attempts to model mortality mechanistically (Sala et al. 2012).

**Figure 2.**
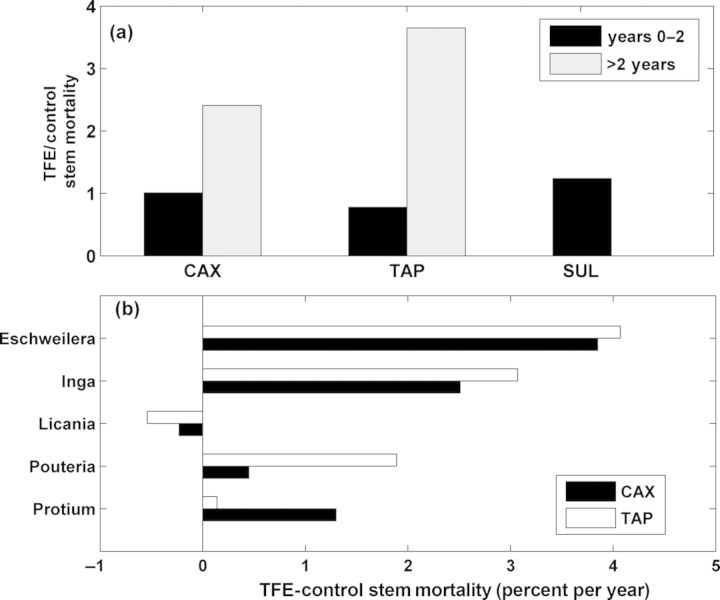
(a) Tree (more than 10 centimeters [cm] diameter at breast height [dbh]) mortality responses in three ecosystem-scale throughfall exclusion (TFE) experiments in tropical rain forest, Caxiuanã National Forest (CAX), Tapajós National Forest (TAP), and SUL (Sulawesi Throughfall Exclusion Experiment; dimensionless quotient of TFE/control). The data are presented for years 0–2 and years 2–7 of TFE treatment (Nepstad et al. 2007, da Costa et al. 2010); the SUL experiment was terminated after 2 years (Moser et al. 2014). The 1-hectare [ha] TFE treatments at CAX and TAP were not replicated because of their large size (Fisher et al. 2007, Nepstad et al. 2007). The TFE design at SUL was three smaller (0.16-ha) plots located close to each other; treatment replication yielded an uncertainty of 1.3% to the mortality response (TFE/stem mortality). Background mortality at CAX was 1.2% ± 0.2 SE (n = 6; da Costa et al. 2010), that at TAP was 2.4% (n = 1; Brando et al. 2008), and that at SUL was 2.1% ±0.4 SE (n = 3, Moser et al. 2014). (b) The absolute difference in tree (more than 10 cm dbh) mortality response to TFE at CAX and TAP for the five most common genera at CAX. The five most common genera at TAP were Eschweilera, Protium, Coussaria, Erisma, and Sclerolobium.

A pantropical analysis of the effects of natural drought on tree mortality (Phillips et al. 2010) used a synthesis of forest plot data (typically 1 hectare [ha]) focused primarily on Amazonia during the 2005 drought in that region, and on Southeast Asia during the 1997–1998 El Niño to suggest a relationship between soil moisture deficit and increased mortality, with both linear and nonlinear relationships between soil moisture deficit and tree mortality considered. Averaged mortality data from two large-scale TFE experiments in Amazonian rain forest were apparently consistent with observations from natural drought at regional scale (Phillips et al. 2010), but the rich longitudinal data and ecosystem process modeling outcomes from these experiments (Fisher et al. 2007, Nepstad et al. 2007, Brando et al. 2008, da Costa et al. 2010) were not explored. More recently, a third large-scale TFE experiment has been reported for Southeast Asia, providing additional insight into the effects of drought in an aseasonal rain forest (Schuldt et al. 2011, van Straaten et al. 2011, Moser et al. 2014). These three soil moisture manipulation experiments are the only such ecosystem-scale experiments reported for tropical rain forest and therefore offer unique insight into the drought–mortality relationship for one of the world's key biomes. Focussing on tree mortality, we compare the results and combine data from these studies to address hypothesis (a).

**Figure 3.**
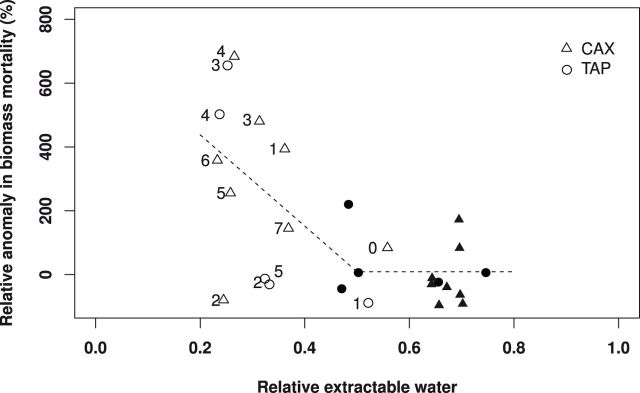
The anomaly in tree (more than 10 centimeters dbh) biomass with respect to background mortality rates at Caxiuanã National Forest (CAX) and Tapajós National Forest (TAP). The triangles represent CAX, the circles represent TAP, the filled symbols represent the control forest, and the open symbols represent throughfall exclusion (TFE)-treated forest. The number adjacent to each open symbol is the year of TFE treatment. The best-fit break point for the piecewise regression fit was at REW = .51. For REW < .51, y = 0.77 + 1404 (0.51 – x) (p < .01); for REW > .51 the slope is not significantly different from zero.

Each of the three ecosystem-scale TFE experiment sites support species-rich, old-growth rain forest, but each differs in annual rainfall and soil characteristics (table 1). The two Amazonian experiments (Meir et al. 2009), in the Brazilian state of Pará in the east of the region, are the ‘Esecaflor’ experiment at Caxiuanã National Forest (CAX); and the ‘Seca Floresta’ experiment at Tapajós National Forest (TAP). Annual precipitation at CAX and TAP is similar at 2000–2300 millimeters (mm), and both experience a significant dry season, with stronger seasonality at TAP, whereas the Sulawesi Throughfall Exclusion Experiment (SUL) in Southeast Asia experiences higher annual precipitation (2900 mm) and almost no seasonality. CAX and TAP are on deep, weathered Oxisols, with CAX having a sandier soil profile (approximately 10 m deep), and TAP having a more clay-rich and very deep (more than 80 m) soil profile; SUL has a well-drained Nitisol (more than 4 m deep). SUL was conducted over 2 years (2007–2009) with continuous rainfall exclusion of 50%–80%. TAP was conducted for 6 years (2000–2005) and CAX is ongoing (since 2001; da Costa et al. 2014). Similar exclusion infrastructure and plot size (1 ha) were used at CAX and TAP, and a similar fraction of incident rainfall excluded (approximately 50%), although at TAP only wet season rainfall was excluded, whereas at CAX, rainfall exclusion was continuous because of the higher risk at that site of dry season storms. The complete suite of measurements and modeling at each experiment varied, but aboveground metrics of tree growth and mortality were recorded at all three sites (Meir et al. 2009, Moser et al. 2014). The responses to TFE can be distinguished by timescale into short-term (0–2 years) and medium-term (3–7 years) effects, although longer-term phases of biomass loss and recovery can also be expected, consistent with basic ecological principles (Shugart 1997, da Costa et al. 2014). The transition from short timescales in the TFE data was characterised by the emergence of substantial changes in tree mortality rates (figures 2 and 3), and at least at one site (CAX), in autotrophic physiology. A summary of responses is provided in box 2, with the mortality data examined below.

**Box 2. Short- and long-term responses by the vegetation to experimental soil moisture deficit.**

**Short-term responses (0–2 years): changes in canopy structure, tree growth and ecophysiology**

The TFE experiment at SUL was imposed for 2 years, whereas at CAX and TAP, the TFE treatment regime continued for several years. There was notable consistency in the response to TFE among sites despite differences in measurement programs. The short-term (0- to 2-year) response by growth to soil moisture deficit was similar, with a reduction in aboveground wood production ranging from 30% to 40% among all three sites. At CAX and TAP, there was a reduction in leaf area index (LAI, m^2^ leaf area per m^2^ ground area) by more than 20% over the first 2 years of the experiment (Meir et al. 2009). At SUL, LAI did not decline, but leaf number per distal branch declined implying a reduction in leaf bud development (Schuldt et al. 2011). The short-term physiological responses in the vegetation were consistent with these reductions in productivity. Maximum leaf photosynthesis was lower at TAP and CAX under soil moisture deficit. At CAX this was shown to be controlled hydraulically: maximum carboxylation capacity did not change significantly but maximum stomatal conductance was down-regulated, with consequent declines observed in sap flux by up to 40% (Fisher et al. 2007, Meir et al. 2009). An increase in the hydraulic resistance of the soil-to-atmosphere water pathway was observed at both CAX and SUL. A combination of soil and plant hydraulics data and soil-to-atmosphere process modeling for CAX indicated that in addition to the stomatal control, the principal locus of increased hydraulic resistance in response to soil moisture deficit was below ground (Fisher et al. 2006, 2007, 2008). At SUL, increased hydraulic resistance was observed in distal twig xylem tissue, with larger trees showing greater reductions in xylem-specific conductivity under soil moisture deficit (Schuldt et al. 2011). At TAP, nocturnal hydraulic redistribution in the root zone was shown to improve soil moisture availability for some trees over the first 2 years, although this process rapidly declined in importance subsequently (Markewitz et al. 2010). At CAX, a multilayer soil and canopy physiology model was developed and parameterised to simulate the response to drought. The model was validated using independent sap flux and soil moisture measurements, and used to quantify responses over the first 2 years of the TFE treatment: stand-level transpiration was estimated to decline by 40%, and gross primary productivity declined by 13%–14% (Fisher et al. 2007).

**A long-term increase in autotrophic respiration**

Only limited longer-term ecophysiological data are available, with a focus on autotrophic respiration at CAX. Leaf dark respiration (*R*_dark_) increased at CAX during the first 7 years of TFE (Metcalfe et al. 2010a), leading to an increase in canopy respiration despite the reduction in LAI. *R*_dark_ has been observed more frequently to decline with photosynthesis, although increases in *R*_dark_ during drought have been noted for other forests, including elsewhere in Amazonia (Metcalfe et al. 2010a). The evidence from measurements of CO_2_ flux from woody tissue and soil at CAX was consistent with this, suggesting that the long-term soil moisture deficit had led to a long-term increase in autotrophic respiration, perhaps in reparatory response to drought stress (Metcalfe et al. 2010b). If photosynthesis in Amazon rain forests declines during drought, and ecosystem respiration does not, there is potential for net ecosystem production (NEP) to switch from a net carbon sink (NEP more than 0) to a source (NEP less than 0; Meir et al. 2008, Rowland et al. 2014). Metcalfe and colleagues (2010b) summed all NPP and respiration terms for control and droughted forest and calculated that total carbon use by the droughted forest likely exceeded GPP. The analysis indicated that the droughted forest had a reduced carbon use efficiency (CUE = NPP/GPP) with respect to control forest, and was potentially acting as a net carbon source (i.e., NEP less than 0), although uncertainty terms were relatively large. New physiological measurements are needed to test the persistence of this finding, quantifying long-term (decadal-scale) responses to the TFE treatment in the key underlying respiratory, hydraulic and photosynthetic parameters.

## Similarity in vegetation responses to extended soil moisture ­deficit?

A surprising degree of similarity in response to the TFE treatments was observed at all three experiments, despite their differing locations, soils, species assemblages and climate. Aboveground wood production (for stems with dbh more than 10 centimeters) declined by 30%–40% (da Costa et al. 2010, Moser et al. 2014) but at no site did tree mortality change for the first 2 years of TFE, suggesting widespread initial resistance in the vegetation to soil moisture deficit (figure 2a). Response data over more than 2 years of TFE are only available from TAP and CAX, but despite the large differences in soil profiles at each site, a consistent pattern emerged, with tree mortality rising rapidly after 2 years of TFE, committing 20%–25% of the original standing biomass (215–270 Mg C per ha) to the atmosphere through future decomposition (Brando et al. 2008, da Costa et al. 2010). Large trees (dbh more than 40 centimeters), which comprised 58%–62% of the original biomass, were the most vulnerable at both CAX and TAP (da Costa et al. 2010), as also observed following natural severe droughts (Phillips et al. 2010), and there was also some phylogenetic consistency among the experiments in terms of the most and least vulnerable taxa. At the family level, the Fagaceae were sensitive to TFE in both Sulawesi and Amazonia (da Costa et al. 2010, Moser et al. 2014). Within the Amazon at both CAX and TAP, the genus *Eschweilera* was very sensitive to the TFE treatment, whereas *Licania* was notably resistant (Nepstad et al. 2007, da Costa et al. 2010); among other taxa the mortality response varied up to fourfold (figure 2b). These data provide unique insight into the long-term composition of tropical rain forest following severe drought. They also offer a focus for efforts to quantify the consequences for ecosystem functioning of drought-related tree mortality, and to test for convergence among drought-tolerant species groups in plant functional traits (Oliveira et al. 2014) and resistance to biotic attack.

Although soil moisture content by volume is commonly measured in ecosystem process studies, it does not quantify the water available to plants because of site-based differences in soil physical properties and plant moisture acquisition strategies (Fisher et al. 2008). To account for this we used a measure of the water available to vegetation, the relative extractable water, REW (box 1). Elsewhere, water deficit metrics for tropical forest regions have been calculated from rainfall estimates and an assumed transpiration rate (e.g., Phillips et al. 2010, Gatti et al. 2014). The REW metric is calculated as a fraction of the maximum water shown to be extractable at each site; therefore, although less easily applicable at a large scale, it provides a more ecologically informative metric by taking account of differences among soils in water availability to plants.

Although the dry season is slightly stronger at TAP than at CAX, there was no marked difference between sites in the year-to-year variability in the minimum observed REW, of 0.2 (figure 3). The mortality responses to REW relative to background rates were very similar, despite large edaphic differences between the sites. Mortality showed no trend when soil moisture availability was high, at REW values greater than 0.5, although there was interannual variability of 100%–200% in background mortality rates (figure 3). However, as REW declined below 0.5, a significant fourfold linear increase in mortality up to 700% was observed, indicative of a new and similar response phase in both forest ecosystems. The mortality increase varied at both sites with the duration of TFE, peaking at 3–4 years, with some decline in large tree mortality after 4–5 years (figure 3; da Costa et al. 2010). Therefore, the effects of soil moisture deficit on tree mortality changed over time at both sites, as noted for temperate systems (e.g., Barbeta et al. 2013); tree size classes were affected differentially, and contingently on the preceding drought or mortality events. Modeling of these longer-term dynamics under drought therefore needs to represent pre- and postmortality processes, as well as tree death. Consistent with this, the longest term growth data from both TAP and CAX, following 6–10 years of TFE, suggest an additional phase of resilience in both ecosystems, with a recovery in LAI or in the growth of the smaller remaining trees (Brando et al. 2008, da Costa et al. 2010, da Costa et al. 2014). Whether a second mortality pulse occurs beyond 10 years of TFE at CAX with substantial effects on ecosystem-scale carbon fluxes, or whether the surviving species assemblage and tree-size distribution is resistant to extended soil moisture deficit, remains an open question.

## Drought-mortality processes in tropical rain forests

The observed mortality response to severe natural drought in the tropics (Meir and Grace 2005, Phillips et al. 2010) has in general been more rapid than in the TFE experiments. This likely reflects the additional stress that occurs during natural drought from the combination of increased maximum vapor pressure deficit and leaf temperatures in addition to soil moisture deficit (although foliar water uptake might have contributed to the slower mortality response in the TFE experiments). Phillips and colleagues (2010) also showed that aseasonal Bornean rainforests appeared to have larger mortality responses to natural drought than their more seasonal Amazonian counterparts. It is not clear whether these regional differences reflect differences in drought severity, soil properties or less well drought-adapted vegetation phenotypes. However, we speculate that at any one site, short term severe natural drought and high temperatures may cause significant hydraulic failure in leaves or leaf mortality, leading to rapid death for vulnerable individuals and taxa. By contrast, the effects of extended soil moisture deficit are likely to be characterized by reduced hydraulic function or a reduction in the availability of carbon resources from photosynthesis, together exposing trees to progressively higher risk from pest or pathogen attack, in addition to the failure in physiological processes. The stand-scale data from CAX show some consistency with the ecophysiological components of this interpretation. Notwithstanding the possibility of species-based variation in hydraulic and carbon-use traits, very strong canopy-scale reductions in hydraulic performance were observed in the short term at CAX (less than 1 year of TFE; Fisher et al. 2006, Fisher et al. 2007), whereas Metcalfe and colleagues (2010b) argued that beyond three years of TFE, as mortality increased, the droughted forest was also expending approximately 7 tons of carbon per ha per year more than it was acquiring through gross primary production, consistent with the notion of a declining reserve of stored carbon.

The TFE experiments inform our understanding of the effects of soil moisture reduction alone on mortality, but they do not necessarily specify the mechanism of tree death (e.g., McDowell et al. 2013). In a recent test of five dynamic global vegetation models using the TAP and CAX data, none replicated well the loss of biomass in response to the long-term TFE, with variance in model predictive skill reflecting the need for better representation of both moisture deficit effects on plant function and phenology, and of biomass loss processes (Powell et al. 2013). Separately, where six vegetation models explicitly representing both hydraulic and carbohydrate flux processes were tested against a two-species TFE experiment in the semiarid southeast United States, both hydraulic and carbon-based processes were implicated in the mortality process (McDowell et al. 2013). A second, more demanding, test would be to model the combined effects of atmospheric and edaphic drivers of severe natural drought stress on tree mortality. Representing the dominant mode(s) of tree death during a drought therefore remains a challenge, especially when the responses to drought may affect mortality risk over several years (Anderegg et al. 2013), creating lags in the drought–mortality relationship and its effects on ecosystem-scale processing of carbon and water. A tractable and physiologically meaningful way of modeling the multiple pathways to mortality may be to define a stress threshold beyond which death becomes likely because of physiological failure or biotic attack. A focus on mechanistically driven estimates of physiological stress leading singly or in combination to a threshold value beyond which mortality increases for given species groups could provide an heuristic and effective way to guide research on drought–mortality responses. Such an approach should be flexible enough to allow for mortality to occur rapidly—as well as after repeated or extended drought events—and therefore remain consistent with evidence from TFE experiments and natural drought events (Phillips et al. 2010, Anderegg et al. 2013, Barbeta et al. 2013). A threshold concept based only on carbon storage has been used in dynamic global vegetation models (e.g., Powell et al. 2013), but a metric that integrates both hydraulic and carbon-related responses, and probably with its principal locus in the leaf, may ultimately prove more flexible with respect to different locations and climate change scenarios; and it may also facilitate detection of increased mortality risk at large scales, via the use of rapidly developing remote sensing techniques (Asner et al. 2011).

## Responses by soil respiration to reductions in soil moisture

Soil respiration (*R*_s_) comprises the largest source of CO_2_ to the atmosphere in the terrestrial carbon cycle and substantial changes in *R*_s_ can alter the sign of net ecosystem productivity (Davidson et al. 2006, Meir et al. 2008). At a single site, the response to soil moisture by *R*_s_ is usually—but not always—peaked, with lower *R*_s_ values at high and low soil moisture content (Davidson et al. 2006). Very wet soils impose diffusion limitations on the supply of oxygen to respiring cells and the surface emissions of CO_2_, whereas at the dry end, cellular desiccation and a reduced supply of soluble carbon act to constrain *R*_s_ (Davidson et al. 2006). The soil moisture value at which the threshold, or maximum, *R*_s_ occurs can vary among sites (Wood et al. 2013) and is usually somewhere near field capacity, when optimal gas and substrate diffusion co-occur. As with representing mortality responses to drought, finding ways to generalize these observations in an appropriate and scaleable manner is an important goal, both to advance understanding and to inform predictive models of ecosystem function.

Experimental TFE in tropical forests where soil processes have been studied has been implemented at five sites in addition to the three ‘ecosystem-scale’ TFE experiments considered above. The study plots have varied in size from 1.5 m^2^ to 1 ha, and the TFE treatments have varied in duration from three months to a decade-long experiment (table 1). The sites are all in tropical rain forest, range in mean annual precipitation from 1800 mm to 5000 mm and have soils that vary in texture and depth. There has been a correspondingly large range in responses by *R*_s_ to the TFE treatment: where soil moisture was successfully reduced, responses in *R*_s_ ranged from no effect to a 48% decline (Cattânio et al. 2002, Sotta et al. 2007, Davidson et al. 2008, Metcalfe et al. 2010b, van Straaten et al. 2011, Wood and Silver 2012). Numerous explanations for the range in responses by *R*_s_ to TFE have been put forward and reflect the diversity of processes contributing to *R*_s_, as well as possible differences in TFE implementation. The range of hypothesised biophysical responses include: differential responses of root, litter, and soil organic matter to soil moisture status (Sotta et al. 2007, van Straaten et al. 2011); differences in soil nutrient availability (Wood and Silver 2012); differences in rooting depth (Davidson et al. 2008); and changes in dissolved organic carbon concentrations together with oxygen availability (Cleveland et al. 2010). We hypothesized that the variability in *R*_s_-moisture responses among studies might be explained more generally by differences in the microscale structure and properties of soil, and the related fine-scale effects on the availability of moisture to microbes and roots. Despite recent advances in quantifying soil physical properties for some tropical regions (e.g., Amazonia; Marthews et al. 2014) there remains a general lack of the soil physics measurements needed to parameterise hydraulics models for the tropics (Fisher et al. 2008). However, soil texture information is widely available and potentially provides a first-order proxy for moisture availability to chemical and biological processes in soil. It forms the focus for our analysis addressing hypothesis (b) (see box 1).

### Influence of soil texture on soil respiration

**Figure 4.**
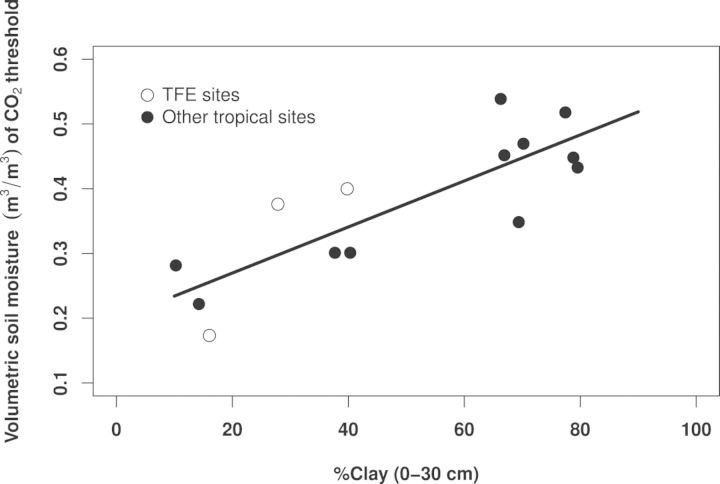
Variation between the percentage clay content and the volumetric soil moisture value of the surface soils (i.e., 0–30 centimeters in depth) at which soil respiration, R_s_, peaks (f = 0.1986 + 0.0036x, n = 14, r^2^ = .70, p < .001). Only three out of eight throughfall exclusion studies reported a soil moisture–R_s_ response function. The data sources from other observational tropical rain forest studies are supplied in appendix S1. Abbreviations: cm, centimeter;  m3/m3, cubic meters of water per cubic meter of soil.

We did not observe any consistent effect of rainfall, temperature, soil moisture, soil nutrient content, or soil texture on the *R*_s_ response to TFE across the eight studies. Only three of the TFE experiments quantified a soil moisture–*R*_s_ response function; however, when combined with additional observational studies from tropical forests (figure 4; also see supplemental appendix S1), we found a strong relationship between the clay content of soil and the threshold soil moisture value at which *R*_s_ peaks (figure 4; *r*^2^ = .70, *p* < .001). Tropical clay soils are variable in gross hydraulic structure and related water-retention properties, but the fine texture and surface characteristics of clay particles also place strong fundamental controls on the sorption and movement of water at the scales of fine root hairs and microbes (Hodnett and Tomasella 2002). Whereas the average pore size of soil decreases with clay content, the number of pores and the water holding capacity increase. If limitation to diffusion occurs at higher soil moisture values in clay soils, this could explain the increase in the soil moisture optimum for *R*_s_ with increasing clay content (figure 4), although the factors determining the observed declines in *R*_s_ from this maximum value in response to TFE appear to be site specific.

Although figure 4 explores the short-term response by *R*_s_ to soil moisture, very few studies have considered the effects of a multiyear soil moisture deficit on *R*_s_. A burst in *R*_s_ is sometimes observed following single rewetting events, but extended drought or a series of dry–rewetting cycles could have a net directional effect on *R*_s_, with acclimation in soil microbes or plant roots. Data from Puerto Rico showed microbial communities that have been pre-exposed to drought conditions to be more resistant to subsequent drought, a result consistent with data from temperate ecosystems showing microbial acclimation to rapid changes in carbon availability (Bouskill et al. 2012). With respect to long term responses in plant respiration (box 2), following 4 years of TFE the *R*_s_ data from CAX showed a much smaller drought-related reduction relative to the large reductions in *R*_s_ observed in the initial 2 years of the experiment (Sotta et al. 2007, Metcalfe et al. 2010b). This reduced *R*_s_ response following extended TFE may have resulted from increased root respiration rates as the trees became more moisture stressed, as was observed in other components of the vegetation (box 2; Metcalfe et al. 2010a, 2010b). On the other hand, short-term (0–2 years) and longer-term (5 years) TFE at TAP had little overall effect on *R*_s_ (Davidson et al. 2008). The lack of a net *R*_s_ response was ascribed in this latter case to the potentially balancing effects of deep root metabolism and root necromass decomposition maintaining *R*_s_, despite the effects of low soil moisture on respiration processes nearer the surface (Brando et al. 2008, Davidson et al. 2008). A recent synthesis of *R*_s_ responses to experimental soil moisture reduction in (mostly) temperate ecosystems demonstrated that equations derived from observations of natural variation in *R*_s_ and soil moisture tended to perform poorly during experimental drought, most probably because changes in soil and root properties under drought were not accounted for in the short-term control data used to determine the empirical models (Vicca et al. 2014). Our analysis, particularly of the longer-term tropical TFE studies, is ­consistent with this and demonstrates the need for improved mechanistic understanding that integrates both carbon inputs to, as well as outputs from the soil during extended changes in climate.

## Synthesis

This analysis partially supports both hypotheses regarding drought-related thresholds in tree mortality and *R*_s_ in tropical rain forests. Figure 3 supports the case for a nonlinear response in tree mortality to soil moisture deficit that is similar across sites (hypothesis (a)). This two-phase pattern of resistance followed by instability may be typical of the initial response by an ecosystem to continuous environmental stress (Scheffer and Carpenter 2003), although we also note the importance of accounting for lagged effects on longer-term ecological dynamics and ecosystem process stability. The soil moisture metric REW offers a simple and ecologically robust way of comparing tropical forest functioning on different soils and under different precipitation regimes, but the relationship with mortality needs to be tested in less seasonal tropical rain forests, such as in Southeast Asia, where soil or vegetation properties may vary substantially, and ideally further afield, in drier climates as well. Collectively, the data from TAP, CAX and SUL are consistent with notions of both hydraulic and metabolic limitation of trees under severe drought, but as for many temperate zone studies, they do not distinguish which process dominates the path to increased tree mortality, or whether increased susceptibility to biotic attack is ultimately the most important effect. This question remains a future challenge. Our second hypothesis (b), that soil texture explains the wide variance in *R*_s_ responses to soil moisture content, was not supported in our TFE synthesis analysis. However, soil clay content was correlated strongly with the threshold soil moisture content for maximum *R*_s_, with clay-rich soils requiring higher water content to yield peak *R*_s_ (figure 4). Given the wide availability of soil texture data, the modulation of peak *R*_s_ in relation to clay content may have general application in constraining models of *R*_s_, depending on model complexity.

Although TFE experiments do not replicate all the climate drivers of a natural drought, by changing only soil moisture status, these large-scale manipulations do enable explicit testing of the soil moisture response functions embedded within ecosystem models (McDowell et al. 2013) and may also reveal unexpected process-level responses (box 2; Meir et al. 2008, Metcalfe et al. 2010a). The size of the treatment effect in such global change experiments has been argued to be smaller and ‘more realistic’ when experiments are conducted over long timescales and when multiple environmental treatments are combined (Leuzinger et al. 2011, Wu et al. 2011, Barbeta et al. 2013). However, the tropical rain forest TFE studies here only partially support this reasoning. For example, although there was a long-term decline in *R*_s_ after 4 years of TFE at CAX (Metcalfe et al. 2010b), the effects of low soil moisture on the mortality of large trees was initially small, then peaked and subsequently declined over 10 years (figures 2 and 3; da Costa et al. 2014). Although some of the TFE results show a degree of consistency with mortality data obtained from natural severe droughts (Phillips et al. 2010), the more rapid path to tree mortality for some tropical trees following natural droughts, where vapor pressure deficit and leaf temperature extremes are combined with soil moisture deficit, also show that multitreatment effects on an ecosystem can be larger, or occur faster, than single-factor effects. Therefore, the combination, severity, and duration of drought events will likely affect different processes such as mortality, autotrophic respiration, and *R*_s_ in different ways. The key is to acknowledge this complexity and use a combination of experimental and observational data to generate strong new model-testing frameworks, focusing on ecological processes and their interactions over different timescales.

## Conclusions

The risk of increased drought in the twenty-first century may be high in some tropical regions (Fu et al. 2013, Reichstein et al. 2013), but the sensitivity of tropical rain forests to drought is substantially higher than in many current dynamic global vegetation models (Galbraith et al. 2010, Huntingford et al. 2013), even before interactions between drought and fire are considered (Brando et al. 2014). The effects of drought on the functioning of a forested ecosystem take place at multiple interacting scales. Understanding and representing this in a sufficiently parsimonious and mechanistic way to enable prediction is a significant challenge for ecological and Earth system science, and is important for the environmental governance decisions which they influence (Meir and Woodward 2010).

Collectively, the eight TFE experiments to date in tropical rain forest show that despite large intersite variation, there is generality in the sensitivity of tropical rain forest trees and soil to significant soil moisture deficit. There are identifiable soil moisture thresholds for tree mortality and *R*_s_ beyond which phases of rapid change can be expected, although wider testing is desirable. Mortality data from TFE experiments and observational studies are strongly consistent in showing high sensitivity to severe drought, especially in larger trees, but there are differences as well. For example, some mortality occurs more rapidly following severe natural droughts, and observational studies do not consider the dynamics resulting from longer-term drought. Integrated model, observational and experimental analysis is needed to probe and represent better the process interdependency causing both short- and longer-term ecological dynamics. However, our experimental data sets still only extend a little beyond a decade and this limits model validation exercises. The long-term TFE experiment data suggest that following rapid mortality among drought-sensitive taxa, some recovery might be expected, with a new and identifiable emergent species assemblage (da Costa et al. 2014). But what happens at multidecadal timescales or longer? This question is pressing, and achieving robust answers will require a combination of approaches in concert with those discussed here, including more use of long-term and spatially extensive observational data (e.g., Fauset et al. 2012) and of paleoecological inference (e.g., Zuidema et al. 2013).

## Supplemental material

The supplemental material is available online at *http://bioscience.oxfordjournals.org/lookup/suppl/doi:10.1093/biosci/biv107/-/DC1*.

## Supplementary Material

SUPPORTING INFORMATION
