# Supplementary material for: Threshold Responses to Soil Moisture Deficit by Trees and Soil in Tropical Rain Forests: Insights from Field Experiments
Source: Bioscience. 2015 Aug 31;65(9):882–92. doi: 10.1093/biosci/biv107 (PMC4777016; doi:10.1093/biosci/biv107)
Supplement: SUPPORTING INFORMATION [file supp_65_9_882__index.html]

Threshold Responses to Soil Moisture Deficit by Trees and Soil in Tropical Rain Forests: Insights from Field Experiments — SUPPORTING INFORMATION 

# Threshold Responses to Soil Moisture Deficit by Trees and Soil in Tropical Rain Forests: Insights from Field Experiments

## SUPPORTING INFORMATION

- Supplemental material.
