# Supplementary material for: Threshold Responses to Soil Moisture Deficit by Trees and Soil in Tropical Rain Forests: Insights from Field Experiments
Source: Bioscience. 2015 Aug 31;65(9):882–92. doi: 10.1093/biosci/biv107 (PMC4777016; doi:10.1093/biosci/biv107)
Supplement: SUPPORTING INFORMATION [file supp_biv107_supplement.docx]

**Appendix S1.** Table of data used in figure. 4. Three of the TFE experiment studies reported a soil moisture threshold for soil respiration, *R*_s_. The remaining tropical rain forest studies, spanning a wider range in soil clay fraction, are observational studies.

| Location | Site Description | VSM of CO_2_ Threshold | % Clay | Citation |
| --- | --- | --- | --- | --- |
| Tambopata, Peru | Sandy soil | 0.280 | 10.0 | 6 |
| Caxiuana, Brazil | Sandy soil | 0.220 | 14.0 | 3 |
| Caxiuana, Brazil* | TFE site, sandy soil | 0.17 | 16.0 | 7 |
| Luquillo, Puerto Rico* | Valley | 0.375 | 27.8 | 5 |
| Caxiuana, Brazil | Clay soil | 0.220 | 38.0 | 3 |
| Manaus, Brazil | INPA Jacaranda plots | 0.300 | 40.0 | 1 |
| Sulawesi, Indonesia* | Lore Lindu National Park | 0.400 | 40.0 | 4 |
| La Selva, Costa Rica | A4 | 0.540 | 66.8 | 2 |
| La Selva, Costa Rica | A3 | 0.450 | 67.6 | 2 |
| La Selva, Costa Rica | L4 | 0.350 | 70.0 | 2 |
| La Selva, Costa Rica | A2 | 0.470 | 70.8 | 2 |
| La Selva, Costa Rica | L5 | 0.520 | 78.0 | 2 |
| La Selva, Costa Rica | L6 | 0.450 | 79.5 | 2 |
| Manaus, Brazil | INPA Reserva Biológica do Cuieiras | 0.440 | 80.0 | 3 |
| * Tropical TFE experiment site | |  |  |  |
|  |  |  |  |  |

*Citations for table*

1. Chambers, J. Q., E. S. Tribuzy, L. C. Toledo, B. F. Crispim, N. Higuchi, J. dos Santos, A. C. Ara√∫jo, B. Kruijt, A. D. Nobre, and S. E. Trumbore. 2004. Respiration from a Tropical Forest Ecosystem: Partitioning of Sources and Low Carbon Use Efficiency. Ecological Applications: 72–88.

2. Schwendenmann, L., E. Veldkamp, T. Brenes, J. O’Brien, and J. Mackensen. 2003. Spatial and temporal variation in soil CO2 efflux in an old-growth neotropical rain forest, La Selva, Costa Rica. Biogeochemistry 64:111–128.

3. Sotta, E. D., E. Veldkamp, B. R. Guimaraes, R. K. Paixao, M. L. P. Ruivo, and S. S. Almeida. 2006. Landscape and climatic controls on spatial and temporal variation in soil CO2 efflux in an Eastern Amazonian Rainforest, Caxiuana, Brazil. Forest Ecology and Management 237: 57–64.

4. van Straaten, O., E. Veldkamp, and M. D. Corre. 2011. Simulated drought reduces soil CO2 efflux and production in a tropical forest in Sulawesi, Indonesia. Ecosphere 2:art119.

5. Wood, T. E., M. Detto, and W. L. Silver. 2013. Sensitivity of soil respiration to variability in soil moisture and temperature in a humid tropical forest. PLoS ONE 8:e80965.

6. Zimmermann, M., P. Meir, M. I. Bird, Y. Malhi, and A. J. Q. Ccahuana. 2009. Climate dependence of heterotrophic soil respiration from a soil-translocation experiment along a 3000 m tropical forest altitudinal gradient. European Journal of Soil Science 60: 895–906.

7. Sotta, E. D., E. Veldkamp, L. Schwendenmann, B. R. Guimaraes, R. K. Paixao, M. d. L. P. Ruivo, A. C. Lola da Costa, and P. Meir. 2007. Effects of an induced drought on soil carbon dioxide (CO2) efflux and soil CO2 production in an Eastern Amazonian rainforest, Brazil. Global Change Biology 13: 2218–2229.
